# Supplementary material for: Quadruple gene-engineered natural killer cells enable multi-antigen targeting for durable antitumor activity against multiple myeloma
Source: Nat Commun. 2022 Nov 29;13:7341. doi: 10.1038/s41467-022-35127-2 (PMC9709157; doi:10.1038/s41467-022-35127-2)
Supplement: Supplementary file 1 — Supplementary Information [file 41467_2022_35127_MOESM1_ESM.pdf]

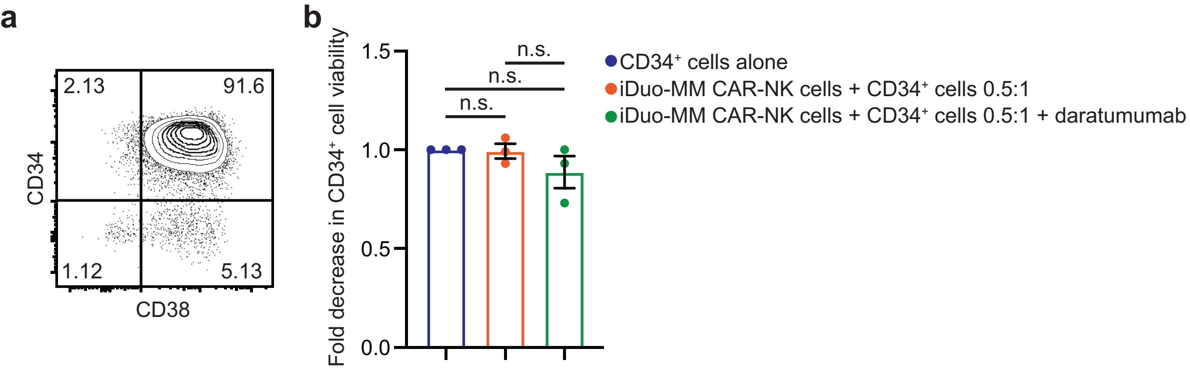


**Supplementary Figure 1. iDuo-MM CAR-NK cells display limited cytotoxicity against CD34⁺CD38⁺ hematopoietic progenitor cells in the presence of daratumumab.** CD34⁺ cells were isolated from umbilical cord blood units (*n* = 3). (a) Representative flow cytometry plot of CD34 and CD38 surface levels on HPCs. (b) CD34⁺CD38⁺ HPCs were labeled with CellTrace dye and co-cultured with iDuo-MM CAR-NK cells at a 0.5:1 E:T ratio in the presence or absence of daratumumbab for 5 hours. The frequencies of HPCs remaining in each culture condition was determined by flow cytometry. Error bars represent mean values with SEM. Statistical significance was determined using two-sided paired Student's *t* tests.

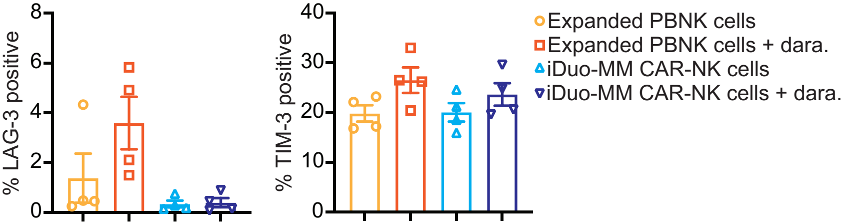


**Supplementary Figure 2. iDuo-MM CAR-NK cells express moderate levels of TIM-3 and very low levels of LAG-3 after after serial restimulation with MM1.S tumor cells.** Serial restimulation assays were performed using NucLight Red-transduced MM.1S cells as targets with 4 independent batches of iDuo-MM CAR-NK cells and expanded PBNK cells isolated from 4 healthy donors at a 3:1 E:T rato in the presence and absence of daratumumab. At the end of the assays, cells were collected and analyze by flow cytometry to assess the frequencies of cells with surface LAG-3 and TIM-3. Shown is cumulative data. Error bars represent means with SEM.

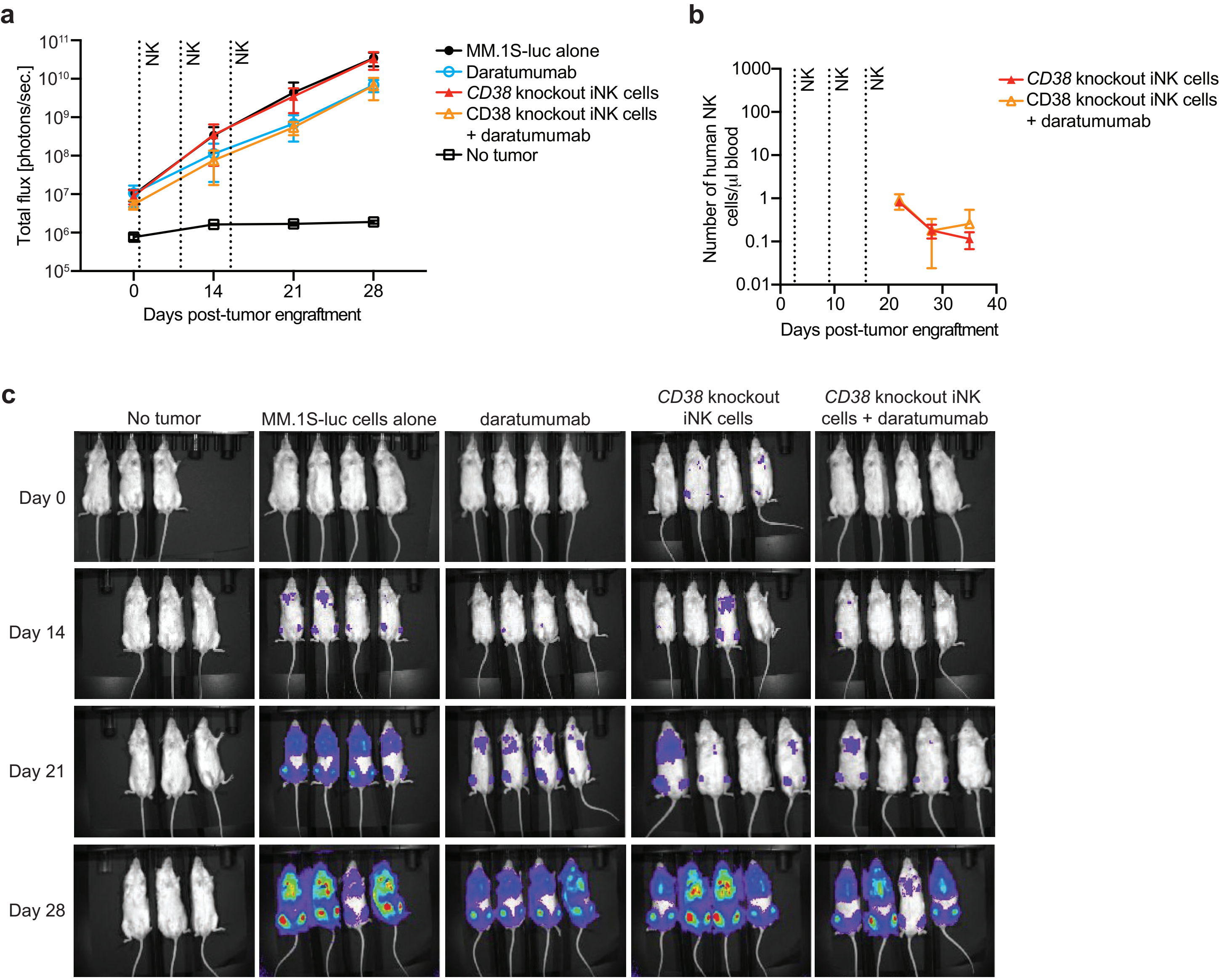


**Supplementary Figure 3. iNK cells with a single *CD38* knockout edit fail to control MM.1S tumor growth and persist in vivo.** NSG mice (*n* = 20) were engrafted with 2x10$^5$ luciferase-transduced MM.1S cells. After 2 days, groups of mice (*n* = 4 group) received no treatment, daratumumab alone, 3 i.v. injections of 1x10$^7$ *CD38* knockout iNK cells, or 1x10$^7$ *CD38* knockout iNK cells + daratumumab. Cells were thawed from cryopreservation and injected immediately into mice. Second and third injections were given at days 9 and 16 post-tumor engraftment. Shown are (a) Graphical representation of BLI data, (b) analyses of human NK cell counts in peripheral blood, and (c) bioluminescence imaging throughout the 4-week experiment. Error bars represent means with SD.

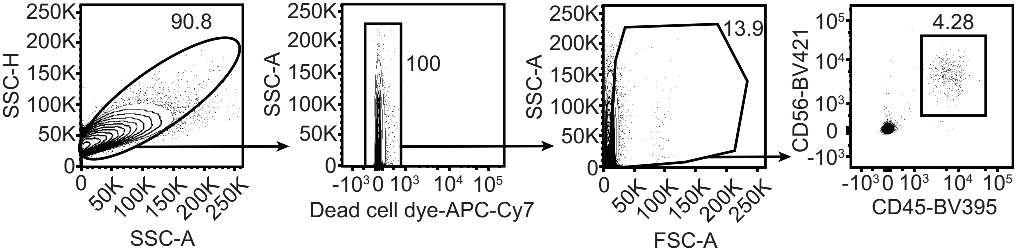


**Supplementary Figure 4. Gating strategy for identification of human NK cells in mouse peripheral blood.** Shown is the full gating strategy used to identify and quantify adoptively transfered NK cells within mouse peripheral blood using flow cytometry for a representative animal.

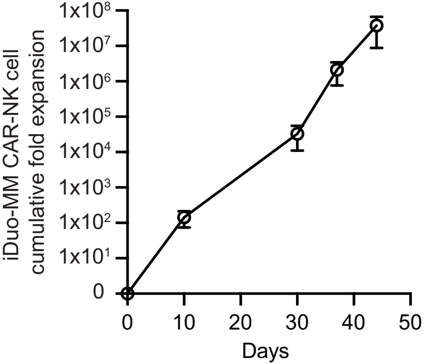


**Supplementary Figure 5. Expansion rates of iDuo-MM CAR-NK cells.** Quadruple-modified iPSCs from 3 independent batches were differentiated to iCD34 cells, further differentiated into iDuo-MM CAR-NK cells, and expanded with engineered K562 feeder cells. Shown is cumulative fold expansion for 3 independent cultures and expansions. Error bars represent means with SD.
