# Supplementary material for: Quadruple gene-engineered natural killer cells enable multi-antigen targeting for durable antitumor activity against multiple myeloma
Source: Nat Commun. 2022 Nov 29;13:7341. doi: 10.1038/s41467-022-35127-2 (PMC9709157; doi:10.1038/s41467-022-35127-2)
Supplement: Supplementary file 3 — Reporting Summary [file 41467_2022_35127_MOESM3_ESM.pdf]

nature portfolio

Corresponding author(s): Jeffrey S. Miller, Bahram Valamehr

Last updated by author(s): November 7, 2022

Reporting Summary

Nature Portfolio wishes to improve the reproducibility of the work that we publish. This form provides structure for consistency and transparency in reporting. For further information on Nature Portfolio policies, see our Editorial Policies and the Editorial Policy Checklist.

Statistics

For all statistical analyses, confirm that the following items are present in the figure legend, table legend, main text, or Methods section.

n/a	Confirmed	
☐	☒	The exact sample size (*n*) for each experimental group/condition, given as a discrete number and unit of measurement
☐	☒	A statement on whether measurements were taken from distinct samples or whether the same sample was measured repeatedly
☐	☒	The statistical test(s) used AND whether they are one- or two-sided<br>*Only common tests should be described solely by name; describe more complex techniques in the Methods section.*
☐	☒	A description of all covariates tested
☐	☒	A description of any assumptions or corrections, such as tests of normality and adjustment for multiple comparisons
☐	☒	A full description of the statistical parameters including central tendency (e.g. means) or other basic estimates (e.g. regression coefficient) AND variation (e.g. standard deviation) or associated estimates of uncertainty (e.g. confidence intervals)
☐	☒	For null hypothesis testing, the test statistic (e.g. *F*, *t*, *r*) with confidence intervals, effect sizes, degrees of freedom and *P* value noted<br>*Give P values as exact values whenever suitable.*
☒	☐	For Bayesian analysis, information on the choice of priors and Markov chain Monte Carlo settings
☒	☐	For hierarchical and complex designs, identification of the appropriate level for tests and full reporting of outcomes
☒	☐	Estimates of effect sizes (e.g. Cohen's *d*, Pearson's *r*), indicating how they were calculated

Our web collection on statistics for biologists contains articles on many of the points above.

Software and code

Policy information about availability of computer code

Data collection	Living Image Software (PerkinElmer), IncuCyte S3 software (Essen BioScience), BD FACSDiva software (BD Biosciences)
Data analysis	FlowJo software (v10.7.1) (BD Biosciences), GraphPad Prism 9 software (GraphPad)

For manuscripts utilizing custom algorithms or software that are central to the research but not yet described in published literature, software must be made available to editors and reviewers. We strongly encourage code deposition in a community repository (e.g. GitHub). See the Nature Portfolio guidelines for submitting code & software for further information.

Data

Policy information about availability of data

All manuscripts must include a data availability statement. This statement should provide the following information, where applicable:

- Accession codes, unique identifiers, or web links for publicly available datasets
- A description of any restrictions on data availability
- For clinical datasets or third party data, please ensure that the statement adheres to our policy

A data availability statement is present, and an excel sheet with source data is included. Hyperlinks with GenBank accession numbers are included in the manuscript.

Human research participants

Policy information about studies involving human research participants and Sex and Gender in Research.

Reporting on sex and gender	N/A
Population characteristics	N/A
Recruitment	N/A
Ethics oversight	N/A

Note that full information on the approval of the study protocol must also be provided in the manuscript.

Field-specific reporting

Please select the one below that is the best fit for your research. If you are not sure, read the appropriate sections before making your selection.

[x] Life sciences [ ] Behavioural & social sciences [ ] Ecological, evolutionary & environmental sciences

For a reference copy of the document with all sections, see nature.com/documents/nr-reporting-summary-flat.pdf

Life sciences study design

All studies must disclose on these points even when the disclosure is negative.

Sample size	No statistical methods were used to determine sample sizes. The size of mouse treatment groups in in vivo studies were determined based on previous studies by our group testing the antitumor function of iPSC-derived NK cells (doi: 10.1126/scitranslmed.aaz5618, doi: 10.1016/j.stem.2021.08.013, doi: 10.1182/blood.2021015184. Equal numbers of male and female mice were used and balanced across groups. For in vitro experiments, sample sizes were determined by how many independent iNK cell differentiations and expansions were available. Basically, most groups had an n of at 3-5, and this was sufficient for determining statistical significance.
Data exclusions	No data was excluded from the manuscript
Replication	All attempts at replication were successful. Most in vitro assays were carried out several times. Because of the size and expense of the in vivo experiments, they were carried out once.
Randomization	In all animal experiments, mice were randomized into treatment groups after tumor was established. Randomization was not necessary for in vitro systems since batches of iNK cells and expanded PBNK cells were being tested with tumor targets. Variables such as sex and age were not relevant.
Blinding	Mice in treatment groups were assigned numbers at random, and the individuals recording BLI data were unaware of which groups received which treatments. Blinding for in vitro experiments was impractical.

Behavioural & social sciences study design

All studies must disclose on these points even when the disclosure is negative.

Study description	N/A
Research sample	N/A
Sampling strategy	N/A
Data collection	N/A
Timing	N/A
Data exclusions	N/A
Non-participation	N/A
Randomization	N/A

Ecological, evolutionary & environmental sciences study design

All studies must disclose on these points even when the disclosure is negative.

Study description	N/A
Research sample	N/A
Sampling strategy	N/A
Data collection	N/A
Timing and spatial scale	N/A
Data exclusions	N/A
Reproducibility	N/A
Randomization	N/A
Blinding	N/A

Did the study involve field work? ☐ Yes ☒ No

Field work, collection and transport

Field conditions	N/A
Location	N/A
Access & import/export	N/A
Disturbance	N/A

Reporting for specific materials, systems and methods

We require information from authors about some types of materials, experimental systems and methods used in many studies. Here, indicate whether each material, system or method listed is relevant to your study. If you are not sure if a list item applies to your research, read the appropriate section before selecting a response.

Materials & experimental systems

n/a	Involved in the study	
☐	☒	Antibodies
☐	☒	Eukaryotic cell lines
☒	☐	Palaeontology and archaeology
☐	☒	Animals and other organisms
☒	☐	Clinical data
☒	☐	Dual use research of concern

Methods

n/a	Involved in the study	
☒	☐	ChIP-seq
☐	☒	Flow cytometry
☒	☐	MRI-based neuroimaging

Antibodies

Antibodies used

anti-CD3, BioLegend, clone: OKT3, Cat#317310, dilution: 50x
anti-CD56, BioLegend, clone: HCD56, Cat#318314, dilution: 50x
anti-DNAM-1, BioLegend, clone: 11A8, Cat#338311, dilution: 50x
anti-NKp44, BioLegend, clone: P448, Cat#325107, dilution: 50x
anti-NKG2D, BioLegend, clone: 1D11, Cat#320805, dilution: 50x
anti-CD16, BioLegend, clone: 3G8, Cat#980112, dilution: 50x
anti-CD38, BioLegend, clone: HIT2, Cat#980308, dilution: 50x

anti-IL-15R, BioLegend, clone: JM7A4, Cat#330207, dilution: 50x
anti-CD138, BioLegend, clone: MI15, Cat#356505, dilution: 50x
anti-BCMA, BioLegend, clone: 19F2, Cat#357517, dilution: 50x
anti-LAG-3, BioLegend, clone: 7H2C65, Cat#369211, dilution: 50x
anti-TIM-3, BioLegend, clone: F38-2E2, Cat#345011, dilution: 50x
anti-CD45, BioLegend, clone: HI30, Cat#304006, dilution: 50x
anti-CD34, BioLegend, clone: 581, Cat#343510, dilution: 50x
anti-NKp30, BD Biosciences, clone: P30-15, Cat#563384, dilution: 50X
anti-KIR2DL1, R&D Systems, clone: FFAB1844P, Cat#FAB1844P-025, dilution: 100x
anti-KIR2DL2/3, R&D Systems, clone: FAB1848P, Cat#FAB1848P, dilution: 100x
BCMA-biotin, AcroBiosystems, clone: BCA-H82E4, Cat#BCA-H82E4-25ug, dilution: 500x

Validation

All antibodies are validated for use in flow cytometry. Data are available on the manufacturer's websites. The antibodies have been validated by the manufacturers. No additional validation was carried out.

Eukaryotic cell lines

Policy information about cell lines and Sex and Gender in Research

Cell line source(s)

MM.1S multiple myeloma cells, MM.1R multiple myeloma cells, RPMI-8226 plasmacytoma cells, Nalm6 acute lymphoblastic leukemia cells, OP9 bone marrow stromal cells, and K562 chronic myeloid leukemia cells were all purchased from the American Tissue Culture Collection (ATCC). All cell lines were maintained in RPMI 1640 media (Corning) supplemented with 10% fetal bovine serum (HyClone), 50 U/ml penicillin/streptomycin, and L-glutamine.

Authentication

Cells were authenticated by the vendor. No other authentications were performed

Mycoplasma contamination

Mycoplasma contamination was tested for monthly. No evidence of contamination was observed.

Commonly misidentified lines
(See ICLAC register)

No commonly misidentified cell lines were used in this study.

Animals and other research organisms

Policy information about studies involving animals; ARRIVE guidelines recommended for reporting animal research, and Sex and Gender in Research

Laboratory animals

Six-to-eight-week-old NSG mice purchased from Jackson Laboratories were used for in vivo studies. Both male and female mice were used.

Wild animals

No wild animals were used.

Reporting on sex

Equal numbers of male and females used in each experiment.

Field-collected samples

No field-collected samples were used.

Ethics oversight

All experiments were reviewed and approved by the University of Minnesota Institutional Animal Care Committee (IACUC) under the protocol 1907-37257A.

Note that full information on the approval of the study protocol must also be provided in the manuscript.

ChIP-seq

Data deposition

☐ Confirm that both raw and final processed data have been deposited in a public database such as GEO.

☐ Confirm that you have deposited or provided access to graph files (e.g. BED files) for the called peaks.

Data access links
May remain private before publication.

N/A

Files in database submission

N/A

Genome browser session
(e.g. UCSC)

N/A

Methodology

Replicates

N/A

Sequencing depth

N/A

Antibodies	N/A
Peak calling parameters	N/A
Data quality	N/A
Software	N/A

Flow Cytometry

Plots

Confirm that:

- [x] The axis labels state the marker and fluorochrome used (e.g. CD4-FITC).
- [x] The axis scales are clearly visible. Include numbers along axes only for bottom left plot of group (a 'group' is an analysis of identical markers).
- [x] All plots are contour plots with outliers or pseudocolor plots.
- [x] A numerical value for number of cells or percentage (with statistics) is provided.

Methodology

Sample preparation	For phenotypic analyses, cells were surface-stained with the indicated antibodies and a fixable dead cell stain in FACS buffer (PBS supplemented with 2% FBS and 2mM EDTA).
Instrument	BD LSRII and Fortessa
Software	FlowJo 10.7.1
Cell population abundance	Cells were counted prior to staining. At least 1 million cells per sample were analyzed by flow cytometry.
Gating strategy	Lymphocytes were initially gated based on forward and side scatter profiles. Live cells were then gated based on exclusion of a dead cell dye. Positive and negative cell populations were gated based on staining the fluorescently-conjugated monoclonal antibodies. Positive and negative staining for all antibodies have been validated using non-stained cells and isotype control antibodies.

- [x] Tick this box to confirm that a figure exemplifying the gating strategy is provided in the Supplementary Information.

Magnetic resonance imaging

Experimental design

Design type	N/A
Design specifications	N/A
Behavioral performance measures	N/A

Acquisition

Imaging type(s)	N/A
Field strength	N/A
Sequence & imaging parameters	N/A
Area of acquisition	N/A

Diffusion MRI ☐ Used ☐ Not used

Preprocessing

Preprocessing software	N/A
Normalization	N/A
Normalization template	N/A

Noise and artifact removal	N/A
Volume censoring	N/A

Statistical modeling & inference

Model type and settings	N/A
Effect(s) tested	N/A

Specify type of analysis: ☐ Whole brain ☐ ROI-based ☐ Both

Statistic type for inference (See Eklund et al. 2016)	N/A
Correction	N/A

Models & analysis

n/a	Involved in the study	
☒	☐	Functional and/or effective connectivity
☐	☒	Graph analysis
☒	☐	Multivariate modeling or predictive analysis

Functional and/or effective connectivity	*Report the measures of dependence used and the model details (e.g. Pearson correlation, partial correlation, mutual information).*
Graph analysis	Statistical analyses were performed using GraphPad Prism
Multivariate modeling and predictive analysis	*Specify independent variables, features extraction and dimension reduction, model, training and evaluation metrics.*
